# Delineating Pro-Angiogenic Myeloid Cells in Cancer Therapy

**DOI:** 10.3390/ijms19092565

**Published:** 2018-08-29

**Authors:** Benjamin W. Johnson, Bhagelu R. Achyut, Sadanand Fulzele, Ashis K. Mondal, Ravindra Kolhe, Ali S. Arbab

**Affiliations:** 1 Department of Pathology, Medical College of Georgia, Augusta University, Augusta, GA 30912, USA; bejohnson1@augusta.edu (B.W.J.); amondal@augusta.edu (A.K.M.); 2 Cancer Animal Models Shared Resource, Winship Cancer Center of Emory University, Atlanta, GA 30322, USA; 3 Department of Orthopedics, Medical College of Georgia, Augusta University, Augusta, GA 30912, USA; sfulzele@augusta.edu; 4 Tumor Angiogenesis Laboratory, Georgia Cancer Center, Augusta University, Augusta, GA 30912, USA

**Keywords:** angiogenic, myeloid, chemotherapy, radiotherapy, immunotherapy, antiangiogenic, MDSC, tumor

## Abstract

Recent evidence suggests that myeloid cells are critical in cancer development and therapy resistance processes. Pharmacological targeting of tumor-associated myeloid cells is an emerging approach among upcoming immune therapies. Surprisingly, myeloid cells are heterogeneous, including a subset of the myeloid cell displaying angiogenic properties in solid tumors. There is an urgent need to delineate angiogenic myeloid cell populations in order to facilitate specific targeting of protumor myeloid cells among heterogeneous pool. This review article is intended to compile all the relevant information in the literature for improved understanding of angiogenic myeloid cells and their role in tumor refractoriness to cancer therapy.

## 1. Introduction

In the normal wound healing process or in cases of acute inflammation, myeloid cells, cells of the innate immune system, are initially recruited to the site of inflammation to clear microbes and debris. Fascinatingly, once the inflammatory signals are primarily resolved, these cells can flip their function to become immune-suppressive and even proangiogenic, to allow for tissue repair. While fascinating and beneficial under normal physiologic conditions, these myeloid cell functions are considered pathologic in cancer because often myeloid cells become immune-suppressive and angiogenic before the inflammation, the malignancy, is resolved. Research has shown that factors released into the tumor microenvironment (TME) epigenetically induce such myeloid cell functions. These myeloid cells ultimately aid in tumor progression and seem to be a significant barrier to cancer therapies, a true testament to the profound effect cancers can have on the physiology of the host.

The heterogeneity of myeloid cell populations in malignancies has proved to be a complication in understanding their roles in tumor progression. Even under normal physiologic conditions, myeloid progenitor cells do not form a clear hierarchical system, but rather a network of cells that can differentiate into various subsets of more-specialized cells [1]. This elusive feature of myeloid cell differentiation persists during their pathological activation in cancers, making these pathological cells challenging to define. Broadly, the pathologic myeloid cell populations that have been detected in tumors can be broken down into two classes: immature myeloid-derived suppressor cells (MDSCs) and tumor-associated myeloid cells (TAMCs), which can still be tumorigenic but are further differentiated. The term myeloid-derived suppressor cell (MDSC) was coined in 2007 in an attempt to describe a collection of immature cells of the myeloid lineage, which are pathologically activated under a chronic inflammatory state and exhibit an immune suppressive phenotype [2]. However, since 2007 many publications have demonstrated that there is phenotypic and functional heterogeneity even within the class of cells referred to as MDSCs. They can be subdivided into monocytic-MDSCs (M-MDSCs), polymorphonuclear-MDSCs (PMN-MDSCs), and early stage-MDSCs (eMDSC) (see [3] for current standards of nomenclature) [3]. TAMCs include tumor-associated macrophages (TAMs), tumor-associated neutrophils (TANs), and tumor-associated dendritic cells (TADCs), all of which can exhibit tumorigenic function [1]. In 2016, Bronte et al. published recommendations for the nomenclature and identification of myeloid cells populations in cancers. They include phenotypic, functional, and biochemical standards by which to identify subpopulations of MDSCs as well as the other tumor-associated myeloid cells. Until an updated set of comprehensive recommendations are published, future research and publications should consider these suggestions for the sake of cohesiveness [3].

All this being said, the most critical concept one must grasp about myeloid cell heterogeneity in cancer is that these cells seem to have an extraordinary level phenotypic and functional plasticity, and there is no clear hierarchy of differentiation. Their differentiation and terminal phenotype and function are dependent on the factors present in the microenvironment, and the epigenetic alterations these factors induce. To illustrate this, it has been shown that immature, pathogenic MDSCs can further differentiate into pathogenic tumor-associated cells (TAMs, TANs, TADCs), or in the presence of the right signaling factors, even be reprogrammed into immunostimulatory neutrophils, monocytes, and dendritic cells [1,4].

As discussed above, the immunosuppressive function of MDSCs and TAMCs is induced by pro-inflammatory cytokines released by the tumor stroma, which signal myeloid cells through a group of well-studied transcription factors: NF-κB, STAT1, STAT3, STAT6, PGE2, and COX2. While M-MDSCs, PMN-MDSCs, eMDSCs, TAMs, TANs, and TADCs all utilize multiple distinct mechanisms of immune suppression, they all act on T cells, and their immunosuppressive mechanisms can be grouped into 4 classes [2]:

1. Depletion of nutrients required by lymphocytes
2. Generation of oxidative stress
3. Interference of lymphocyte trafficking and viability
4. Activation and expansion of T_reg_ cell populations

More recently, the “endoplasmic reticulum (ER) stress response” has been indicated as a driver of the immune suppressive activity of myeloid cells [2]. “ER stress” is a state of disturbed protein folding capacity of the ER, which is induced by conditions associated with tumors: nutrient deprivation, hypoxia, oxidative stress, etc. ER stress triggers an evolutionarily conserved response termed the unfolded protein response (UPR). The UPR is a sequential, triphasic response in which cells make transcriptional changes to: (1) adapt to the changed environment by enhancing ER folding capacity; (2) trigger alarm by inducing transcription of host defense proteins, and lastly; and (3) trigger apoptosis [5]. The current belief is that once myeloid cells have been recruited to the tumor stroma, the tumor microenvironment induces in them a state of ER stress, triggering the UPR. The UPR involves upregulation of proteins such as X-box binding protein 1 (XBP1), and its activating enzyme, inositol-requiring enzyme 1α (IRE1α), as well as C/EBP homologous protein (CHOP). Evidence suggests that UPR proteins like CHOP and XBP1 modulate myeloid cell activity in tumors in a way that activates immunosuppressive function via mechanisms that are not yet entirely clear [6].

Now that we have shed a brief light on the heterogeneity of myeloid cells and their immune suppressive functions, it is time to discuss another side to myeloid cell involvement in cancer, angiogenesis. In 2004, Yang et al. first demonstrated the tumor pro-angiogenic function of immune suppressive Gr1^+^ CD11b^+^ cells in a murine model. Briefly, let us note that Gr1 and CD11b are mouse markers that do not distinguish between PMN-MDSCs, which are immature and exclusively tumor-promoting, and TANs, which are further differentiated and can be either tumor-promoting or tumor-suppressing, depending on which cytokines are in the environment [3]. They observed that mouse tumors, co-injected with Gr1^+^ CD11b^+^ cells, exhibited increased vascular density, vascular maturation, and decreased necrosis. Additionally, they were able to offer support that the ability of these cells to promote angiogenesis in tumors was via their ability to produce high levels of matrix metalloproteinase 9 (MMP9). Secondly, they offered evidence that these mouse myeloid cells can acquire endothelial cell properties in the TME and could directly incorporate into tumor endothelium [7]. Although, since the publication of this study, it has been shown that the incorporation of myeloid cells into the tumor endothelium likely does not occur, as they most likely congregate around tumor vasculature. It is important also to note that these cells’ ability to produce high levels of MMP9 not only has significance in angiogenesis, but also in another hallmark of malignancy, invasion, and metastasis. Indeed, TAMs have been shown to aid in local invasion of malignant cells by secreting matrix metalloproteinases [8]. Since the findings of Yang et al. in 2004, many mechanisms of angiogenesis have been demonstrated in MDSCs and tumor-associated myeloid cells. These will be discussed in the present review.

With support for both immune suppressive and pro-angiogenic functions of myeloid cells in cancer, it is no secret why they have received so much attention in the literature over the past 15 years. However, while there are a number of published studies and reviews that detail the mechanisms and significance of immunosuppressive myeloid cells, the literature on angiogenic myeloid cells in tumors is thinner. Therefore, it will be the purpose of the present review to carefully examine the pro-angiogenic mechanisms of myeloid cells in cancer and the role these cells play in therapy-resistant cancers.

## 2. Pro-Angiogenic Myeloid Cells

### 2.1. Heterogeneity of Pro-Angiogenic Myeloid Cells

TAMs, TANs, and MDSCs have all been studied as drivers of tumor angiogenesis. There has also been evidence of non-myeloid cells such as fibroblasts, platelets, and lymphocytes contributing to tumor angiogenesis [9]. The critical thing to note when studying tumor angiogenesis is that it is not beneficial to ascribe it to a single cell type (although we can say that a significant contribution comes from myeloid cells), as cells from the tumor stroma as well as cells recruited to the TME contribute to new vessel growth. It is better to identify cell surface markers and secreted factors that give a cell the ability to drive angiogenesis. Although there are a couple of critical cell-surface markers that can detect a myeloid cell as angiogenic, there is no single marker or single combination of markers that perfectly describes an angiogenic myeloid cell. As it will be discussed below, myeloid cells can employ unique mechanisms to facilitate tumor angiogenesis, and the angiogenic markers and secreted factors that have been mentioned in the literature reflect independent angiogenic mechanisms. Some reliable cell surface markers that can be detected using immunohistochemistry and indicate that a myeloid cell exhibits angiogenic capacity include VEGFR2, VE-cadherin, CD31 (platelet endothelial cell adhesion molecule 1), and TIE2 (angiopoietin receptor) [10,11].

### 2.2. Pro-Angiogenic Mechanisms of Myeloid Cells

Under steady state conditions, angiogenesis is regulated by a balance of secreted pro- and anti-angiogenic factors. Some such proangiogenic factors include vascular endothelial growth factor (VEGF), fibroblast growth factor (FGF), and angiopoietin II (Ang2). Conversely, antiangiogenic factors include thrombospondin, endostatin, and angiostatin. A gradient of proangiogenic factors will result in branching off of new vasculature and growth directed towards the concentration of proangiogenic factors.

It is well recognized that a necessary step in the transition from benign hyperplasia to a solid malignancy is the induction of tumor vasculature, and this event has officially been coined the “angiogenic switch”. The angiogenic switch can be thought of as the point at which the balance between pro- and antiangiogenic factors tips in favor of pro-angiogenic factors, and in contrast to normal wound healing, this imbalance never resolves. This continuous pro-angiogenic signaling leads to the formation of irregular, or ‘messy’ vasculature. The vessels in tumors are leaky and often hemorrhagic, features that aid in tumor cell intravasation and, as we will discuss below, is a barrier to cancer therapy. The blood flow through these vessels is sluggish, and the vessels themselves are irregularly shaped and deficit in perivascular cells, known as pericytes [9]. The idea of an angiogenic switch begs the question, what leads to this imbalance of angiogenic factors? The key regulator of the angiogenic switch seems, logically, to be hypoxia [12]. Hypoxia triggers the release of pro-angiogenic factors from the tumor stroma and leads to the recruitment of other cells namely myeloid cells, which then further facilitate angiogenesis. Hypoxia-induced expression of certain chemokines and proangiogenic factors, CXCL12 (stromal derived factor-1α/SDF-1α) and ANGPT2, respectively, have been shown to recruit angiogenic TAMs [11]. An early study identified MMP9 as a key trigger of the angiogenic switch, and as we have already mentioned, pathologic myeloid cells in the TME are abundant sources of secreted MMP9. In fact, this same study observed that MMP9 was not expressed in tumor cells. Instead, it was expressed in a small population of cells surrounding the vasculature, where angiogenic myeloid cells are known to congregate [13]. Some specific mechanisms by which myeloid cells contribute to the angiogenic switch and sustain tumor angiogenesis will now be discussed.

#### 2.2.1. VEGF-A/VEGFR2

A very well studied pro-angiogenic signaling axis is the vascular endothelial growth factor A-vascular endothelial growth factor receptor 2 (VEGFA-VEGFR2) axis, and indeed, angiogenic myeloid cells in the TME utilize this mechanism. As a matter of fact, VEGFA was initially purified from fluid secreted by a tumor [14]. It has been shown that deletion of VEGF in TAMs delayed the angiogenic switch, suggesting that myeloid cells in the TME directly secrete VEGFA [11]. Yet, as Yang et al. noted in 2004, the increase in VEGFA observed with co-injection of immunosuppressive myeloid cells did not seem to primarily be from direct secretion of VEGFA by myeloid cells, but rather from release by the extracellular matrix (ECM) upon degradation by MMP9, which is abundantly secreted by these myeloid cells [7]. Once in the extracellular environment, VEGFA binds to the angiogenic receptor, VEGFR2, on nearby endothelial cells (EC). VEGFR2, a tyrosine kinase receptor (TKR), signals through the PLCγ-PKC-MAPK pathway and promotes endothelial cell proliferation [14].

In addition to activating VEGFR2 on endothelial cells (ECs), VEGFA also binds to VEGFR2 on myeloid cells. One study demonstrated that upregulation of VEGFR2 in myeloid cells was necessary for the malignant progression of gliomas in a murine model. Furthermore, deficiency of VEGR2 in murine bone marrow-derived cells suppressed the differentiation of myeloid cells and compromised their angiogenic function. These findings suggest that VEGFA-VEGFR2 signaling in myeloid cells promotes their differentiation and possibly increased secretion of pro-angiogenic factors [15]. VEGFA also has been shown to act on monocytes via another one of its receptors, VEGFR1. Stimulation of VEGFR1 on monocytes facilitates migration of these cells, implicating VEGFA in the recruitment of pathologic myeloid cells to the TME [11,14]. In summary, aside from its action on ECs, VEGFA can facilitate angiogenesis through its activation of VEGFR1 and VEGFR2 on myeloid cells. Activation of these signaling axes promotes differentiation of myeloid cells from the bone marrow, recruitment of these cells to the TME and induction of their pro-angiogenic function.

#### 2.2.2. VE-Cadherin & CD31

Vascular endothelial cadherin (VE-cadherin) is traditionally an EC marker and is recognized as the dominant adhesion molecule responsible for maintaining EC contacts in the vasculature. Interestingly enough, this cadherin protein has been shown to be expressed by cells of the myeloid lineage: CD34^−^/CD14^+^ human peripheral blood monocytes and mouse Gr1^+^ Cd11b^+^ MDSCs/TANs [16]. Similarly, CD31 (platelet/endothelial cell adhesion molecule 1), another traditionally endothelial cell-surface protein that plays a role in EC junctions, has been observed on myeloid cells in tumors. The presence of these EC junction proteins on myeloid cells has in part led to a debatable conclusion that myeloid cells contribute to tumor angiogenesis by directly incorporating into the vascular endothelium or differentiating into ECs that then contribute to the tumor endothelium. However, other studies have refuted this concept. One such study observed that myeloid cells are recruited to the EC wall of blood vessels during angiogenesis, but do not differentiate into ECs or incorporate into the endothelium [17]. This finding suggests that VE-cadherin and/or CD31 expressing myeloid cells could utilize these cell-surface proteins to congregate around existing vasculature by interacting with corresponding receptors on endothelial cell surfaces. If these same myeloid cells also secrete pro-angiogenic factors and/or matrix degrading proteins, expression of VE-cadherin/CD31 would make them very efficient and potent stimulators of angiogenesis.

#### 2.2.3. TIE2

TIE2 is another traditionally endothelial cell receptor that is expressed on myeloid cells in tumors and contributes to their angiogenic ability. TIE2 is a TKR, like VEGFR2, that binds to angiopoietin I and II (ANG1 & ANG2). Broadly, it is thought that ANG1 activation of TIE2 on endothelial cells promotes vascular stability while ANG2 serves as a competitive antagonist to ANG1, inhibiting its angiostatic effects and thereby promoting EC destabilization and angiogenesis. However, recent evidence has shown that ANG1 activation of TIE2 can also promote EC migration and proliferation if it is anchored alone in the ECM, giving TIE2 two conflicting roles of promoting vascular stability and even vascular growth [18].

While primarily studied in the context of endothelial cells, TIE2 is also expressed on myeloid cell populations, namely macrophages. TIE2-expressing macrophages (TEM) are a highly angiogenic subpopulation of TAMs, which are derived from circulating TIE2-expressing monocytes that are recruited to the TME and their expression of TIE2 subsequently upregulated [11,19]. Knockdown of TIE2 expression on TEMs specifically was sufficient to drive vessel regression in tumors [11]. How does then TIE2 expression on myeloid cells give them pro-angiogenic capacity? One study found that a blockade of ANG2 while having no effect on the recruitment of TEMs to the TME decreased their interaction with angiogenic blood vessels and reduced tumor angiogenesis [19]. It is reasonable to think that similar to VE-cadherin and CD31 expressing myeloid cells, TIE2 allows myeloid cells to interact with angiogenic vessels closely. The ECs of these sprouting vessels secrete abundant amounts of ANG2, which seems to sequester TIE2-expressing myeloid cells. These myeloid cells can then further aid in vessel growth, perhaps by secreting matrix-degrading enzymes and pro-angiogenic factors.

#### 2.2.4. Secreted Factors

Thus far, we have focused on four cell surface proteins, VEGFR2, VE-cadherin, CD31, and TIE2, which have all been shown to contribute to the pro-angiogenic capacity of myeloid cells. Said proteins can also be used to identify these cells using IHC. However, their expression of cell surface receptors is not the whole story behind pro-angiogenic myeloid cells. Just like hypoxic cells of the tumor stroma and destabilized ECs, myeloid cells secrete a number of pro-angiogenic factors. An extensive list of these factors includes some that have been discussed in this review and others that are discussed elsewhere: VEGFA, FGF2, tumor necrosis factor α (TNFα), transforming growth factor beta (TGFβ), platelet-derived growth factor (PDGF), placental growth factor (PIGF), neuropilin-1, CXCL chemokines (CXCL-8,12), semaphorins, and some matrix degrading enzymes such as matrix metalloproteinases (MMP-2,7,9,14), and cysteine cathepsin proteases [11]. In particular, research suggests that their ability to secrete matrix-degrading enzymes like MMPs and cathepsins are paramount to their pro-angiogenic capacity.

In summary, oncogenic processes create a TME that is exceptionally metabolically demanding. Rampant oxygen consumption creates a hypoxic environment that triggers malignant cells and cells of the tumor stroma (non-malignant cells associated with and surrounding the tumor) to release pro-angiogenic factors that begin to act on nearby ECs. Cytokines, chemokines, and some of these pro-angiogenic factors recruit circulating myeloid cells with angiogenic capacity. Cell surface proteins, including VE-cadherin, CD31, and TIE2, lead to sequestration of pro-angiogenic myeloid cells around the budding endothelium. Angiogenic receptors, like VEGFR2, respond to pro-angiogenic factors in the TME and promote proliferation of these pro-angiogenic myeloid cells and their own release of pro-angiogenic factors, namely matrix-degrading enzymes. These myeloid cells, although only a fraction of the cells in the tumor stroma, seem to be strong support staff in a tumor’s ability to induce angiogenesis. This can probably be attributed to maximally efficient localization around the endothelium and their ability to secrete sufficient levels of enzymes and factors that aid in angiogenesis. A more detailed list of myeloid cell proteins that give these cells a pro-angiogenic capacity is included in Table 1.

## 3. Pro-Angiogenic Myeloid Cells and Cancer Therapy Resistance

Since the discovery of myeloid cell contribution to tumor progression, multiple studies have demonstrated a correlation between myeloid cell presence in tumors and poor patient outcomes. Furthermore, data exists that suggests that the presence of myeloid cells in cancer significantly contributes to resistance to cancer therapies [4,20]. Despite excellent initial results to different cancer treatment modalities today, it is all too common that initial cancer regression is only transient. In fact, it is so common that it has been suggested that therapeutic resistance could be added as a cancer hallmark [21]. Here we will discuss what role, if any, the angiogenic capacity of myeloid cells in tumors contributes to cancer refractoriness to chemotherapy, radiotherapy, immunotherapy, and antiangiogenic therapy.

### 3.1. Chemotherapy

Chemotherapy, along with radiotherapy, is termed a “cytotoxic therapy” because of its function to kill rapidly dividing cells. Conventional chemotherapy is still used today as the first line of treatment against many cancers, while the precise mutations underlying the cancer are analyzed. Aside from killing massive amounts of tumor cells, chemotherapies also function by inducing collapse of the tumor vasculature, and refractoriness to such therapies largely depends on revascularization and remodeling of the tumor ECM to re-establish a favorable environment for cell growth [22]. One study found that high dose Taxol, fundamental chemotherapy, leads to accumulation of TAMs in mouse models and human breast cancer patients, and these TAMs secreted high levels of cathepsins, which were potent suppressors of cell death induced by Taxol [22]. Cathepsins are ECM degrading enzymes, and as we have discussed, the ability of myeloid cells to produce such enzymes seems to be paramount to their pro-angiogenic function. Indeed, serine and cysteine cathepsins have specifically been implicated in a pro-angiogenic role [9]. This suggests that chemotherapy can change the TME in a way that recruits myeloid cells, which subsequently dulls the efficacy of chemotherapy at hand. 

Another factor that needs to be considered is the delivery of chemotherapeutic agents to the tumor site. As we have discussed, due to the over-expression of the pro-angiogenic signals, tumor vasculature is abnormal, and blood flow is slow, and this blunts the delivery of chemotherapeutic drugs. Deletion of myeloid-derived VEGFA was shown to improve chemotherapeutic drug delivery by normalizing the tumor vasculature. It also improved chemotherapy-induced tumor cell senescence, clearance of senescent tumor cells by natural killer cells, and inhibits tumor regrowth after chemotherapy in a mouse model [23]. Other studies have shown that TIE2-expressing macrophages, a pro-angiogenic myeloid subpopulation that we have highlighted above, promote tumor revascularization and relapse after chemotherapy in both mouse and human tumors. While these effects in TIE2-expressing myeloid cells were in part through their secretion of VEGFA, conditional deletion of TIE2 in macrophages significantly prohibited blood supply and regrowth of tumors after chemotherapy in a mouse model, highlighting TIE2’s specific importance [24,25]. In summary, pro-angiogenic myeloid cells are often recruited to the TME after chemotherapy and contribute to tumor regrowth and refractoriness to chemotherapies by promoting tumor revascularization and inhibiting drug delivery via formation of abnormal tumor vasculature. 

### 3.2. Radiotherapy

Radiotherapy, another form of cytotoxic therapy used for solid tumors, utilizes high doses of either external or internal radiation, often gamma radiation, to damage tumor cell DNA to the extent that the cells either stop dividing or die. Similar to chemotherapy, radiotherapy is usually effective for initial tumor regression, yet recurrence of the primary tumors is the leading cause of death of patients treated with radiation, and myeloid cell infiltration undoubtedly plays a role in this disturbing statistic [26]. Myeloid cells, notably TIE-2 expressing myeloid cells, rapidly accumulate in tumors after local irradiation, and this accumulation seems to be due to the expression of stromal derived factor-1α (SDF-1α), or CXCL12, a factor that released by stromal cells in response to hypoxia [27,28]. This is a response that adheres to the body’s normal physiologic function of recruiting innate immune cells in response to tissue damage. Administering an inhibitor of SDF-1α’s receptor, CXCR4, immediately after radiation treatment significantly delayed tumor regrowth, although this was ineffective when administered 5 days after radiation treatment, attesting to the rapid recruitment of myeloid cells [27].

Furthermore, enhancement of the antitumor response to radiation treatment was observed with administration of CD11b antibodies in a xenograft model of squamous cell carcinoma, providing a causal link between myeloid cells and tumor regrowth following radiation. The aforementioned study demonstrated that CD11b^+^ Gr1^−^ cells, mouse M-MDSCs and TAMs, are even more causally involved in this relationship than CD11b^+^ Gr1^+^ cells, aka mouse PMN-MDSCs and TANs [29]. It should be cautioned, however, when considering a xenograft model, which relies on the response of a murine immune system that the human immune response could vary in the degree and composition of myeloid cell recruitment. Nonetheless, the data is highly suggestive that radiation treatment leads to extensive recruitment of myeloid cells to the tumor and that these cells, monocyte-derived cells in particular, facilitate tumor regrowth after radiation. So how do myeloid cells achieve this? Initial tumor vessel growth, which has been discussed extensively above, along with the substantial majority of vessel growth in the healthy human body, rely on angiogenesis, or the sprouting of new vessels off of pre-existing ones. However, it has been shown that radiation treatment inhibits local angiogenesis, a phenomenon for which there is not a precise mechanism. After radiotherapy, tumors must rely on a “backup” method of novel vessel growth, vasculogenesis, or recruitment of circulating endothelial progenitor cells (EPCs) and formation of brand new vessels, to regrow their blood supply [28,29]. Kozin et al. showed that post-irradiation tumor vasculogenesis seems to be even more dependent on recruitment of angiogenic myeloid cells as compared to EPCs [27]. Together, angiogenic myeloid cells and EPCs reform an irradiated tumor’s vasculature, allowing the tumor to regrow.

### 3.3. Antiangiogenic Therapy

Antiangiogenic therapy (AAT) targets the VEGFA/VEGFR2 axis to inhibit recruitment of tumor vasculature and aid in tumor regression. Thus, the effects of angiogenic myeloid cells on tumor refractoriness to AAT are, as you might expect, very significant. They are reviewed extensively in the literature, so only a brief review will be included here. Generally, AAT has proven effective in human patients, but these results are typically transient. While the VEGFA/VEGFR2 is a potent angiogenic axis, and one indeed employed by angiogenic myeloid cells, it is not the only angiogenic mechanism expressed in myeloid cells. As we have discussed above, there are numerous other cell surface proteins and secreted pro-angiogenic factors that can be employed. Thus, AAT typically leads to recruitment of other angiogenic myeloid cells that utilize VEGF-independent angiogenic pathways, which then facilitate refractoriness to AAT [30]. These results are a testament to the heterogeneity of angiogenic mechanisms in myeloid cell populations.

Moreover, long-term AAT results in vessel blocking or inhibition of vessel growth. As promising as this may seem theoretical, this further starves the tumor of oxygen and accentuates its hypoxic, but not ischemic, environment, which as we have noted is a primary regulator of the accumulation of angiogenic and immune suppressive myeloid cells. Furthermore, the initial beneficial effects seen with AAT could be attributed to vessel normalization (rather than vessel blocking), in turn decreasing tumor hypoxia and lifting the immunosuppressive and angiogenic qualities of such an environment [31]. The distinction between vessel blocking and vessel normalization has significant implications in the effectiveness immunotherapies, which we will now discuss.

### 3.4. Immunotherapy

Immunotherapies all function by assisting the host’s immune system in targeting tumor cells. Immunotherapy is a heterogeneous classification of cancer therapies that achieve their “immune assistance” through different means, but fundamentally all depend on T cell function to detect and destroy tumor cells. We have briefly discussed above, and it is extensively discussed in the literature, that MDSCs and TAMCs recruited to the TME are potent immunosuppressors and seem to function specifically through inhibiting T cell function in both an antigen-specific and -nonspecific manners. One can accurately assume then that myeloid cells are significantly intertwined in the issue of resistance to immunotherapies. Here, however, we will examine what role the angiogenic capacity of myeloid cells plays in resistance to immunotherapies.

A direct link between angiogenic myeloid cells and immunotherapy resistance exists and is centered around our good friend VEGFA, which in addition to promoting angiogenesis, has been shown to inhibit immunity via multiple mechanisms. VEGFA binding to VEGFR1, as opposed to VEGFR2, on CD34+ hematopoietic progenitor cells, inhibits their terminal differentiation into dendritic cells and interferes with their antigen-presenting capacity. It also has been shown to induce expression of programmed death ligand-1 (PDL1), a molecule understood to inhibit T cell activation, in dendritic cells. Additionally, it impedes T cell extravasation by limiting T cell adhesion to the lumen of vessels, inhibits the proliferation of cytotoxic T lymphocytes (CTLs) and stimulates the proliferation of T_reg_ cells [11,23]. Seeing as angiogenesis and immunosuppression are both such well-documented and potent functions of myeloid cells in tumors, it is unsurprising that there exists a direct link between the two.

An indirect connection between pro-angiogenic myeloid cells and immunotherapy resistance exists and is a function of the “messy” vasculature induced by overexpression of pro-angiogenic factors, which is in part due to expression and enhanced bioavailability regulated by angiogenic myeloid cells. In our discussion of chemotherapies, we touched on how the inefficient blood flow through tumor vasculature is a barrier to drug delivery, and this same principle plays a role in the delivery of immunotherapies, such as monoclonal antibodies. Furthermore, the inefficient blood flow through these vessels accentuates the hypoxic environment of the TME. Hypoxia in the TME has been demonstrated extensively to facilitate an immunosuppressive environment, in part through recruitment of immunosuppressive myeloid cells. Indeed, the merit of vessel normalization of tumor vasculature is now being realized. One study showed that hyperoxygenation of the TME increases CTL activity and correlates with improved clinical responses to immune checkpoint inhibitors [31]. Research has shown promising results of combining immunotherapies with strategies to normalize, not block, tumor vasculature to attenuate the hypoxic TME. Such approaches seem to lift the inhibitory effects of a hypoxic microenvironment that could have on T cells. Seeing as angiogenic myeloid cells play such a critical role in the overexpression of pro-angiogenic factors, targeting these cells could be a useful strategy to achieve vessel normalization and improve the efficacy of immunotherapies.

## 4. Summary

A myeloid cell family is a group of fascinating, dynamic, and heterogeneous innate immune cells, which can be immunostimulatory and phagocytic at one time, and immunosuppressive and pro-angiogenic at another. Their phenotype and function are based on secreted factors in their microenvironment and the epigenetic changes that these factors induce. Malignancies recruit myeloid cells to the TME and secrete factors which induce pro-angiogenic and immunosuppressive myeloid cell phenotypes, which aid in their progression.

The potently hypoxic environment created by the high metabolic demand of a malignant tumor causes cells of the tumor stroma to release chemokines and pro-angiogenic factors that recruit circulating myeloid cells that express pro-angiogenic markers, such as VE-cadherin, CD31, VEGFR2, and TIE2. Once in the TME, hypoxia upregulates these pro-angiogenic proteins. Myeloid cell surface proteins like VE-cadherin, CD31, and TIE2 give these cells the ability to localize around pre-existing or newly forming vessels in the tumor. This incredibly efficient localization makes myeloid cells potent facilitators of tumor angiogenesis. Once confined to the tumor vasculature, myeloid cell secretion of pro-angiogenic factors and matrix-degrading enzymes both induces EC proliferation and also degrades the ECM, which makes way for sprouting vessels and releases VEGFA. A complete list of proteins that contribute to the pro-angiogenic capacity of myeloid cells is included in Table 1.

Initial tumor regression following therapy and subsequent tumor regrowth and refractoriness to said therapy is so common that it has been suggested as a hallmark of cancer. Much data exists that indicates that angiogenic myeloid cells play a significant role in refractoriness to chemotherapies, radiotherapies, AATs, and immunotherapies. It has been shown that cancer therapies enhance myeloid cell recruitment to the TME, at which point, pro-angiogenic myeloid cells assist in tumor regrowth by employing their dynamic list of pro-angiogenic mechanisms to facilitate regrowth of tumor blood supply. Additionally, pro-angiogenic myeloid cells play a role in the formation of the abnormal vasculature seen in tumors. This slow, tortuous and leaky tumor vasculature provides another barrier to cancer therapies by both impeding delivery of therapeutic molecules and accentuating a hypoxic TME. A summarized table of myeloid cell contributions to therapy refractoriness is included in Table 2.

## 5. Future Perspectives and Directions

Future studies should continue to provide clarity and maintain synchrony as to the nomenclature and characterization of myeloid cell populations in cancer. The recommendations made by Bronte et al. are currently the most up to date standards of such nomenclature and should be referred to by scientists publishing data about myeloid cells in cancer [3].

Any research into tumor angiogenesis should certainly account for myeloid cell contributions, as they have been shown to be potent and efficient facilitators in the recruitment of tumor vasculature. Future research into therapies that target the tumor vasculature should work towards a goal of tumor vessel normalization, as opposed to vessel inhibition. Tumor vessel normalization attenuates the hypoxic TME, lifting much of the immunosuppressive and pro-angiogenic effects of such an environment. Targeting pro-angiogenic myeloid cells could be a good avenue to explore to achieve tumor vessel normalization.

## Figures and Tables

**Table 1:** Myeloid cells employ a number of different mechanisms to facilitate angiogenesis. Specific cell surface proteins allow myeloid cells to congregate efficiently around budding endothelium. Secreted factors include some matrix-degrading enzymes, which degrade the extracellular matrix (ECM), effectively clearing the way for budding vasculature and releasing vascular endothelial growth factor A (VEGFA) from the matrix. Lastly, myeloid cells secrete pro-angiogenic factors that stimulate endothelial cell (EC) migration and proliferation.

Sequestration Proteins	Matrix Degrading Enzymes	Pro-Angiogenic Factors
VE-cadherin (cell surface)	MMP-2,7,9 and 14 (secreted)	VEGFA (secreted)
CD31 (cell surface)	Cathepsin B (secreted)	FGF2 (secreted)
TIE2 (cell surface)		TNFα (secreted)
		TGFβ (secreted)
		PDGF (secreted)
		Neuropilin-1 (secreted)
		CXCL-8,12 (secreted)
		Semaphorin-4D (secreted)

**Table 2:** Myeloid cell contributions to cancer therapy refractoriness.

Chemotherapy	Radiotherapy	Antiangiogenic Therapy	Immunotherapy
Facilitate angiogenic-revascularization Formation of abnormal tumor vasculature-impede drug delivery, accentuate hypoxic environment	Facilitate vasculogenic-revascularization	Facilitate VEGFA-independent angiogenic-revascularization	Secretion of VEGFA Formation of abnormal tumor vasculature-impede drug delivery, accentuate hypoxic environment

## Abbreviations

TME	Tumor microenvironment
TAMC	Tumor-associated myeloid cell
M/PMN/eMDSC	Monocytic/polymorphonuclear/early-stage–myeloid-derived suppressor cell
UPR	Unfolded protein response
CHOP	C/EBP homologous protein
XBP1	X-box binding protein 1
IRE1α	Inositol-requiring enzyme
MMP	Matrix metalloproteinase
VEGFR2	Vascular endothelial growth factor receptor2
CD31	Platelet endothelial cell adhesion molecule1
TIE2	Tyrosine kinase with immunoglobulin and EGF-like domains
VEGF	Vascular endothelial growth factor
FGF	Fibroblast growth factor
ANG2	Angiopoietin 2
CXCL12	C-X-C motif chemokine 12
SDF-1α	Stromal derived factor-1α
EC	Endothelial cell
TEM	TIE2-expressing macrophage
EPC	Endothelial progenitor cell
AAT	Antiangiogenic therapy
